# Characterization of visceral leishmaniasis outbreak, Marsabit County, Kenya, 2014

**DOI:** 10.1186/s12889-020-08532-9

**Published:** 2020-04-05

**Authors:** Evalyne Wambui Kanyina

**Affiliations:** 1 Kenya Field Epidemiology and Laboratory Training Program, Nairobi, Kenya; 2 Ministry of Health, Nairobi, Kenya

**Keywords:** Visceral leishmaniasis (VL), Kala-azar, VL case management, Marsabit County, Kenya

## Abstract

**Background:**

Visceral leishmaniasis (VL) is caused by protozoa of the *Leishmania donovani* complex. Annually, an estimated 500,000 cases of VL are reported globally posing a public health challenge. The objectives of our study were to confirm and determine the magnitude of VL outbreak, characterize the outbreak clinically and epidemiologically and evaluate the county preparedness and response in Marsabit County, Kenya.

**Methods:**

A retrospective review of laboratory registers and patients’ clinical notes was done at Marsabit County Hospital. Cases were persons with confirmed VL diagnosis either by microscopy, serology or molecular technique coming from Marsabit County from May to October 2014. Cases were interviewed using structured questionnaire to collect clinical and epidemiologic information. Blood samples were collected from cases for laboratory confirmation.

**Results:**

A total of 136 cases were confirmed of which 77% (105) were male with a median age of 17 (IQR: 22) years and 9.6% (13) case fatality rate. All cases were admitted at Marsabit County Referral Hospital, Kenya. Medical records of 133 cases were retrieved. Of the 133 cases, 102 (77%) presented with fever, 43 (32%) with splenomegaly, 26 (20%) with hepatomegaly and 96 (72%) were managed with Sodium stibogluconate (SSG) monotherapy. Thirty-four cases (26%) received Full haemogram (FHG) test and none had more than one Liver Function Tests (LFTs) in a span of 6 months. Presenting with headache (OR: 4.21, 95% CI: 1.10–16.09) and hepatomegaly (OR: 4.2, 95% CI: 1.30–14.11) were associated with VL death. No VL case management training had been conducted nor VL treatment guidelines distributed among health care workers (HCWs) in the last 1 year.

**Conclusions:**

VL cases were confirmed. Inadequate case monitoring and management was evident. VL case management sensitization training was conducted. The County health department should put in place one health VL surveillance and facilitate periodic case management trainings.

## Background

Visceral leishmaniasis (VL), also known as Kala-azar, still remains a public health problem with both human and veterinary health significance. Globally, an estimated 500,000 cases of visceral leshmaniasis occurs annually and 90% of these cases come from six countries - Bangladesh, Brazil, Ethiopia, India, Nepal and Sudan (Leishmaniasis control in Eastern Africa: Past and present efforts and future needs. Situation and gap analysis [6]). In Africa, VL is endemic in five countries; Ethiopia, Kenya, Uganda, Somali and Sudan. In Kenya, it is estimated that about 4000 cases occur annually while 5 million people are at risk of infection (Technical guidelines for Integrated Disease Surveillance and Response in Kenya [20]).

There are two forms of leishmaniasis identified in Kenya; visceral leishmaniasis (VL) and cutaneous leishmaniasis (CL). Visceral leishmaniasis is the prevalent (Leishmaniasis control in Eastern Africa: Past and present efforts and future needs. Situation and gap analysis [6]). The first documented VL cases in Kenya were reported from Wajir and Mandera Counties back in 1935 [2] while the first CL case was described in 1969 (Visceral leishmaniasis is the prevalent (Leishmaniasis control in Eastern Africa: Past and present efforts and future needs. Situation and gap analysis [6]). The two diseases are found in different geographical areas; VL in arid, low-lying areas of the Rift Valley, Eastern and North Eastern provinces (now referred to as Counties) and CL in semi-arid lowlands to high plateaus in the Eastern, Rift Valley, Central and Western provinces (now referred to as Counties) [18]. Additionally, areas around the Rift Valley escarpments and major mountains in Kenya are documented natural habitats for sandflies [11, 16, 21].

Although kala-azar has been detected in both domestic (cattle, dogs, donkeys, goats and sheep) and wild animals [4, 9], little zoonotic studies has been done in this population in Kenya. Their contribution in kala-azar epidemiology is also unclear [4, 9]. Though Kala-azar is curable, it still causes high morbidity and sometime death in humans due to its low index of suspicion by health care providers, late diagnosis and poor cases management. If left untreated, it has a high mortality rate (over 95%) [1].

In May 2014, reports of 18 laboratory (rK39) confirmed Kala-azar cases in Marsabit County from 10th to 21st May 2014 were received at the Ministry of Health through Disease Surveillance and Response Unit (DSRU). This prompted the Ministry of Health to conduct an investigation in Marsabit County. The aim of the investigation was to confirm and determine the magnitude of an outbreak, describe the epidemiology, clinical characteristics and treatment outcomes of rK39 confirmed VL cases, and evaluate the county preparedness and response to the outbreak.

## Methods

### Study design and setting

This study characterizes VL outbreak cases in Marsabit County Referral Hospital, Marsabit County about 550 km North of Nairobi, Kenya. Marsabit is the largest county in Kenya covering 70, 961 square Kilometres. It is divided into 7 Sub-Counties: Marsabit, Laisamis, North Horr, Loiyangalani, Sololo, Moyale and Chalbi. Marsabit borders three counties; Wajir to the east where VL is endemic, Turkana to the west and Isiolo to the south. It also borders the country of Ethiopia to the north. The people of Marsabit County are mainly nomadic pastoralists rearing cattle, goats, sheep and camels. Herding is usually done by men; boys, youths and adults. During the dry period, pastoralists move from one area to another and settle in areas called Fora in search of pasture and water for their livestock. A fora is a forestry and swampy grazing areas where the pastoralists stay with their animals for months before returning to their households. In the fora’s, they sleep in open areas next to the animals without use of mosquito nets.

### Characteristics of participants

All VL cases in the County were referred to this health facility for management. A case of VL was defined as any person living in or has traveled to Marsabit County and complains of fever ≥38^0^ C (or history of fever) or headache for more than 2 weeks and/or splenomegaly, lymphadenopathy, general weight loss, anorexia with VL diagnosis by rK39 antigen-based rapid diagnostic kit; the DiaMed IT-Leish (DiaMed AG, Switzerland), from May to October 2014. DiaMed IT-Leish rk39 rapid diagnostic test (RDT) kits are simple, field friendly and the results are available within 25 min. Additionally, they have a high sensitivity (97–100%) and specificity (97–100%) [3, 14, 15, 19].

### Data collection

Retrospective record review was conducted from May 2013 to May 2014 to determine the period when the outbreak could have started. All health care facilities which had reported at least one suspected VL case were selected for the investigation; Marsabit Sub-County Hospital, Logologo Health Centre and Mountain Clinic. This was followed by prospective record review of medical and laboratory records of VL confirmed cases in Marsabit County Referral Hospital from May 2014 to October 2014. Interviews and data abstraction from medical and laboratory records were conducted using a structured questionnaire (Additional file 1) to collect clinical and epidemiologic information. Variables of interest were epidemiological characteristics: Age, gender, residence, occupation, date seen at the facility, date of admission, date of referral, place of referral and hospitalization days; clinical characteristics: fever ≥38^0^ C (or history of fever), headache, splenomegaly, lymphadenopathy, general weight loss, anorexia, vomiting, poor appetite, anaemia, intermittent respiratory infections and epistaxis; treatment regimen: Sodium stibogluconate (SSG), paramomycin (PM) and liposomal amphotericin B (Ambisome) drugs; biological markers for monitoring treatment: total blood count, kidney and liver function test and treatment outcome: dead or alive.

Approximately 4–6 ml of blood was obtained from consenting or assenting suspected and confirmed cases and stored in a refrigerator at 2-8 °C at Marsabit Sub-County Hospital Laboratory. Assent was sought from all study participants below 18 years before study participation. Samples were centrifuged within 6 h of sample collection. The refrigerator’s temperature was monitored with a thermometer to ensure the temperature stayed within 2–8 °C. Samples were later triple packaged in a cooler box and transported to National Public Health Laboratory services (NPHLs), Nairobi, Kenya by air (Mission Aviation Fellowship) for further differential analysis using Polymerase Chain Reaction (PCR) and Enzyme-Linked Immune-sorbent Assay (ELISA). The results were E-mailed from NPHLs to Marsabit Sub-County Hospital at the completion of testing of all the samples for patient notification and optimal case management. Preparedness and response to VL outbreak was assessed by administering a structured questionnaire (Additional file 2) to the members of the Sub-County and County Health Management Teams through face to face interview. The questionnaire addressed the following areas; information on outbreak preparedness and response, management of information, case management, laboratory surveillance, vector control activities, documentation and data utilization. Verification of responses, where necessary, was made by observation.

### Data analysis

Data generated was entered and analysed using Microsoft Excel 2010. Descriptive statistics were determined. They include measures of frequency (Count, proportions, frequency, rate), measures of central tendency (median) and measures of dispersion (interquartile range). The association between age and clinical presentations of VL cases and disease outcome were determined using odds ratio test.

## Results

### Descriptive investigation of Kala-azar

**Fig. 1:**
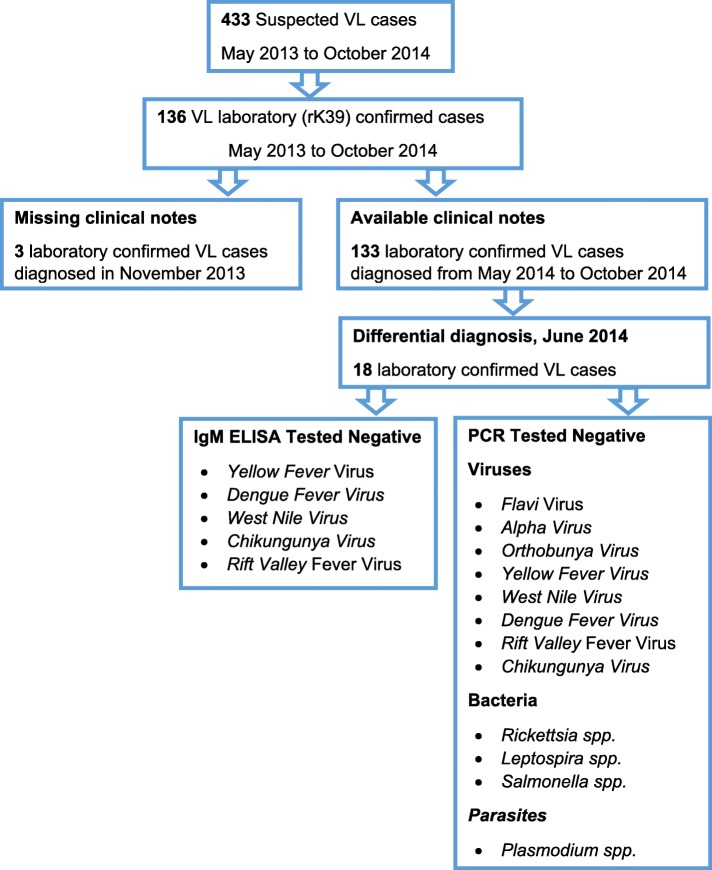
Flowchart of visceral leishmaniasis cases*,* Marsabit County Referral Hospital, Kenya, 2014. Four hundred thirty-three suspected visceral leishmaniasis (VL) cases were reported from May 2013 to October 2014. Of the 433 VL cases, 136 cases tested positive with rK39 test kit. However, only 133 VL cases clinical notes were available for review during the study period. On the other hand only 18 confirmed VL cases underwent differential diagnosis to rule out possible co-morbidities and or other causes of febrile illness

**Fig. 2:**
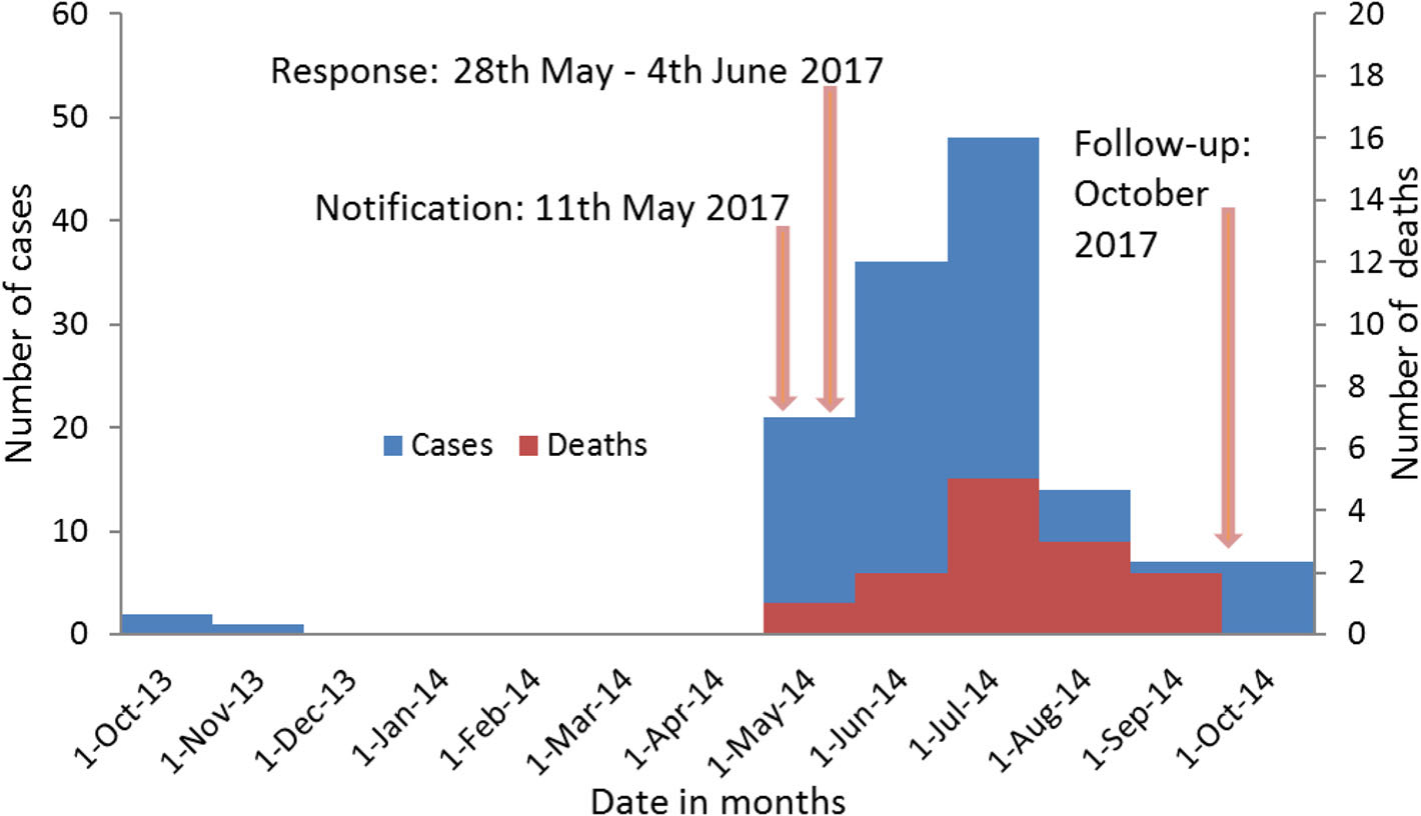
Epicurve of visceral leishmaniasis cases*,* Marsabit County Referral Hospital, Kenya, 2014 *n* = 136. The index case was reported in October 2013. On 11th May 2014, the Ministry of Health was notified of 18 laboratory (rK39) confirmed visceral leishmaniasis cases in Marsabit County from 10th to 21st May 2014. This prompted the Ministry of Health to conduct field investigation to confirm the existence of an outbreak and epidemiologically characterize the outbreak in Marsabit County. New cases continued being seen at the various facilities within the county after visceral leishmaniasis sensitization. Between May and September 2014, 118 visceral leishmaniasis cases have been reported in Marsabit County Hospital of which 12 (10%) cases have been lost to death. In the month of October 2014, follow up investigation was conducted to update the line list, clinically characterize the cases, and describe regimens used to manage the cases and their treatment outcome

**Fig. 3:**
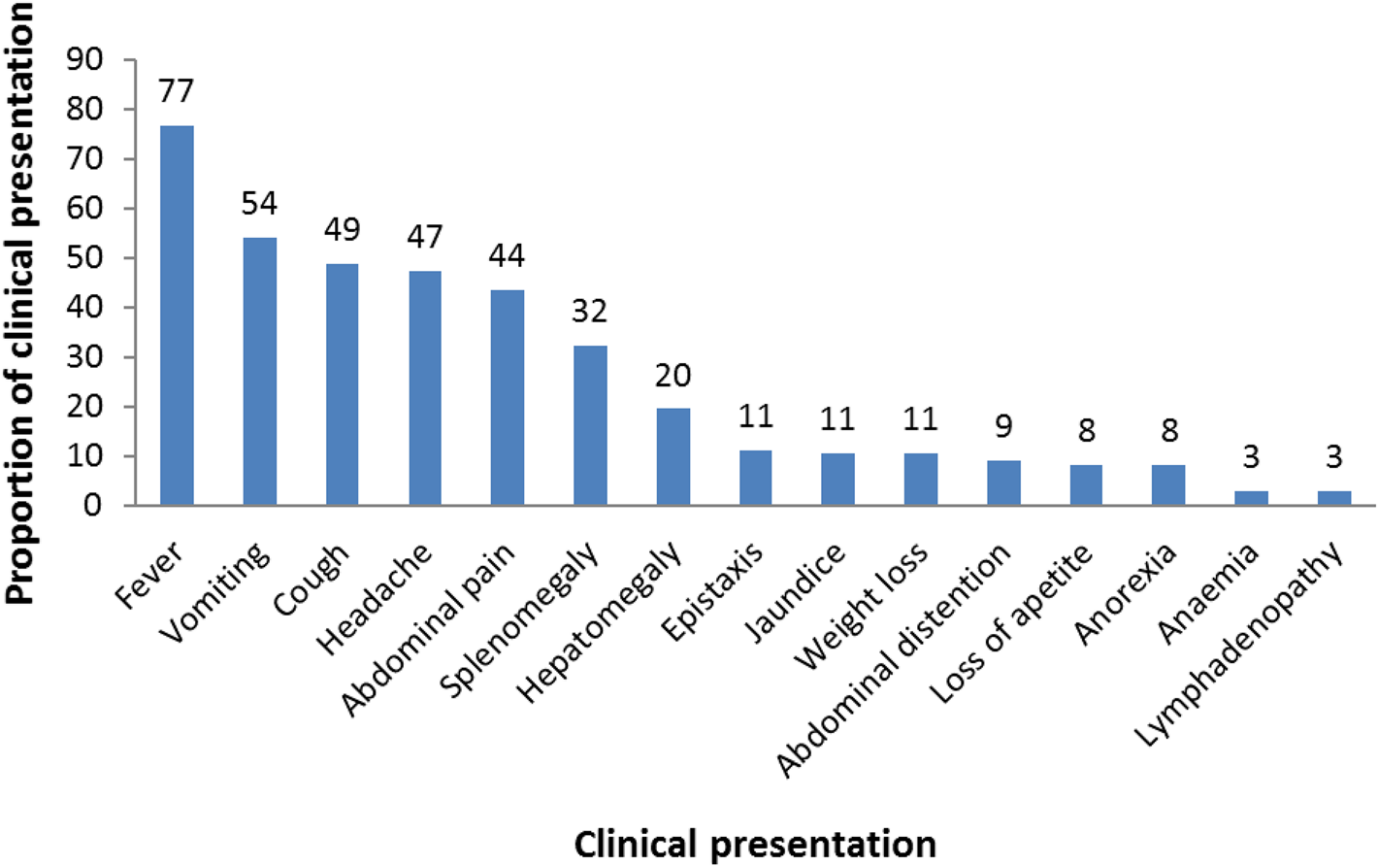
Clinical presentation of visceral leishmaniasis cases*,* Marsabit County Referral Hospital, Kenya, 2014 *n* = 133. These are the clinical presentation of visceral leishmaniasis cases reported in Marsabit County. All the cases upon diagnosis were hospitalized in Marsabit County Referral Hospital for directly observed treatment

A total of 433 suspected VL cases were identified of which 136 (31.4%) were laboratory (rK39) confirmed during the period of the record review (Fig. 1 and Fig. 2). Of the 136 confirmed cases, 105 (77%) were male and the median age was 17 (IQR: 22) years (Table 1). All cases were admitted at Marsabit County Referral Hospital for management. Thirteen deaths were reported (CFR: 9.6%). Two thirds of the confirmed cases came from Bubisa (29%, 40 of 136), Logologo (24%, 33 of 136) and Shurr (13%, 18 of 136) villages. Clinical notes of three confirmed cases diagnosed in November 2013 were missing. Of the 133 confirmed cases with clinical notes, 102 (77%) presented with fever, 72 (54%) with vomiting, 65 (49%) with cough, 63 (47%) with headache, 58 (44%) with abdominal pain, 43 (32%) with splenomegaly and 26 (20%) with hepatomegaly (Fig. 3).

**Table 1:** Age distribution of visceral leishmaniasis cases*,* Marsabit County Referral Hospital, Kenya, 2014 *n* = 145

Age in Years	Frequency	Percentage
Under 5	32	22
5 to 14	36	25
15 to 29	43	30
30 to 44	18	12
45 and above	16	11
**Total**	**145**	**100**

**Fig. 4:**
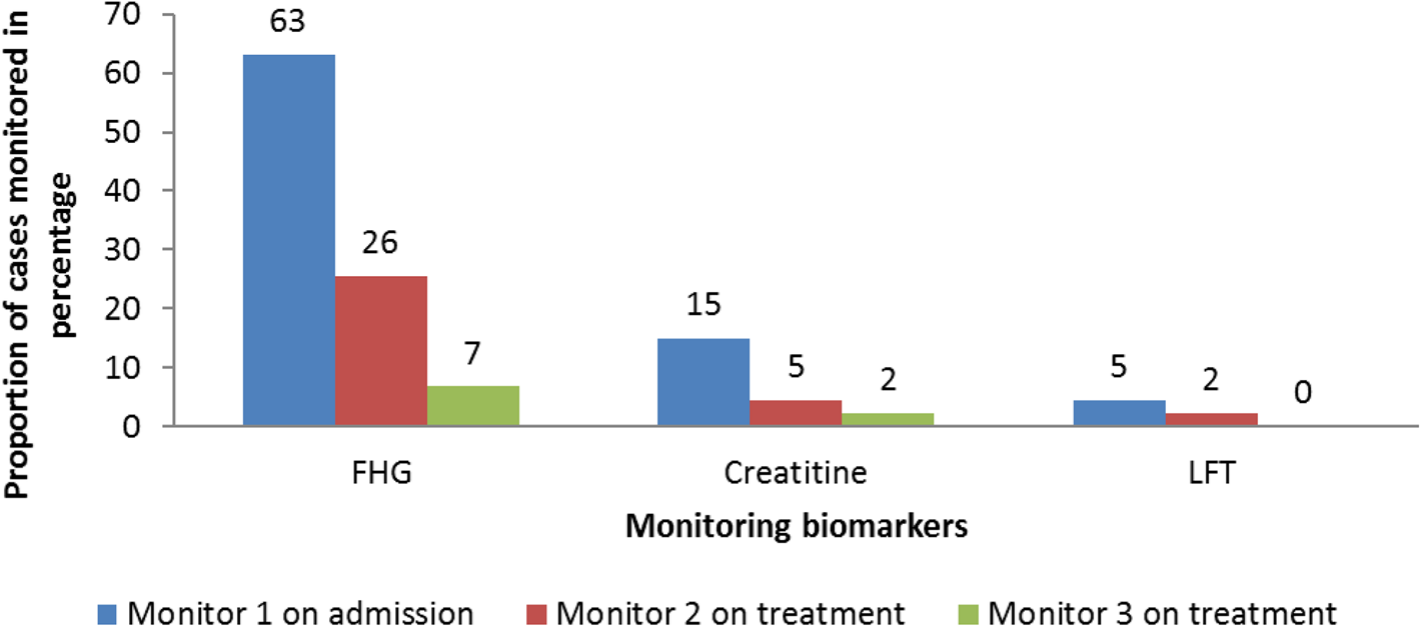
Biomarkers monitored in visceral leishmaniasis cases management*,* Marsabit County Referral Hospital, Kenya, 2014 *n* = 133. Visceral leishmaniasis cases progress on treatment was monitored using various biomarkers namely full haemogram (FHG), kidney function test (creatinine levels) and liver function test. These tests were performed on admission before treatment initiation and during treatment. This shows inconsistent in monitoring of biomarkers

Of the 133 cases admitted, 96 (72%) were treated with Sodium stibogluconate (SSG) monotherapy and 37 (28%) with combined regimen; sodium stibogluconate and paromomycin. Various biomarkers; Full Haemogram (FHG), kidney function test (creatinine levels) and Liver function test, were used to monitor cases’ progress during treatment. Upon admission, 84 (63%) cases had FHG test, 20 (15%) had creatinine levels estimated and six (5%) had liver function test conducted. One week after treatment initiation, 34 (26%) cases had FHG test, six (5%) had creatinine levels estimated and three (2%) had liver function test conducted (Fig. 4). Of the monitoring biomarkers, 34 (26%) cases had at least two Full Haemogram (FHG) tests, seven (5%) cases had at least two creatinine tests and none had more than one Liver Function Tests (LFTs) by the third week of treatment. Ten (8%) cases were transfused with blood.

**Fig. 5:**
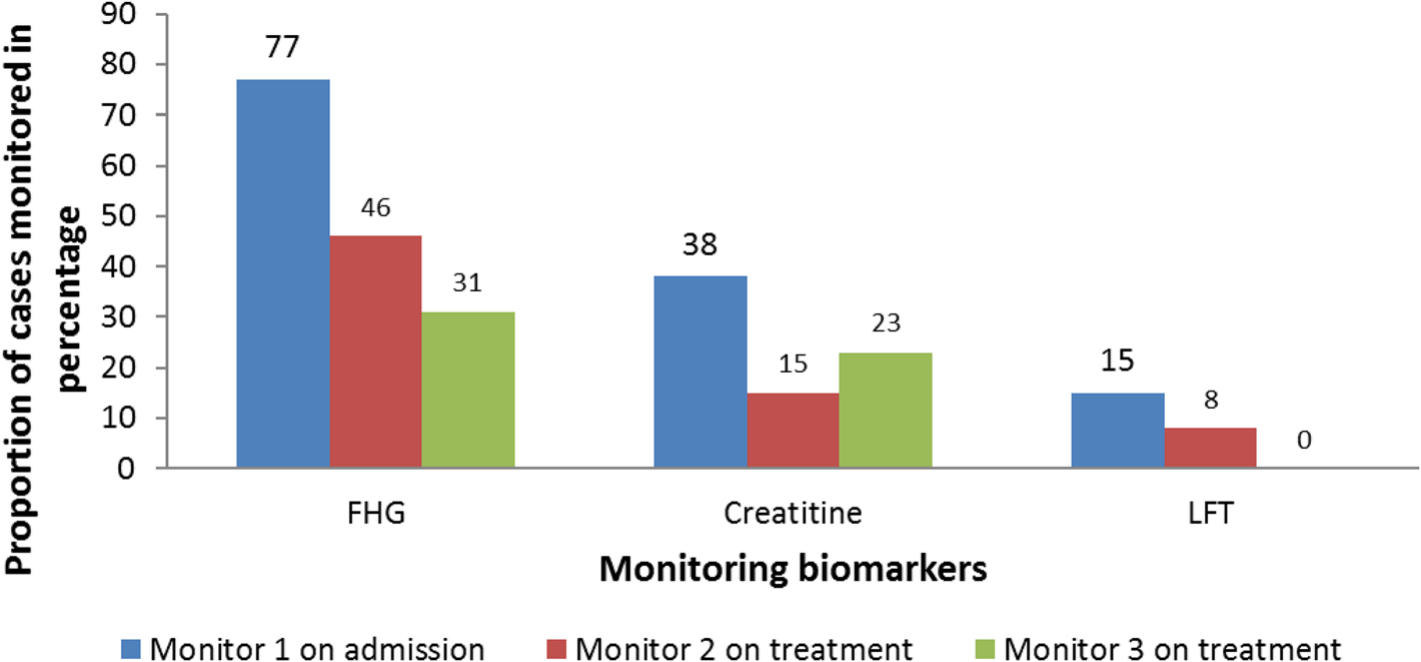
Biomarkers monitored among diseased visceral leishmaniasis cases*,* Marsabit County Referral Hospital, Kenya, 2014 *n* = 13. This is the clinical picture of biomarkers presentation among the diseased visceral leishmaniasis cases. This illustrates that majority of the cases did not receive any form of monitoring

Of the 13 death reported, 12 (92%) were male with a median age of 40 (IQR: 20) years. Cases presented with fever (85%), headache (77%), vomiting (69%), abdominal pain (62%), cough (54%), splenomegaly (46%), hepatomegaly (46%), jaundice (23%), epistaxis (15%), loss of appetite (8%) and abdominal distention (8%). Ten (77%) were treated with Sodium stibogluconate monotherapy and three (23%) were on the combined regimen of sodium stibogluconate and paromomycin. On admission, 10 (77%) cases had FHG test, five (38%) had creatinine levels estimated and two (15%) had liver function test conducted (Fig. 5). A significant number did not receive any form of monitoring; three (23%) had zero FHG test, eight (62%) had zero creatinine test and 11 (85%) had zero LFT test. One (8%) case had blood transfusion.

Bivariate analysis was performed to determine any possible association. Being under-5 years was not associated with VL death (OR: 0.2, 95% CI: 0.03–2.17). However, presenting with headache (OR: 4.21, 95% CI: 1.10–16.09) and hepatomegaly (OR: 4.29, 95% CI: 1.30–14.11) were associated with VL death.

### Laboratory analysis

A total of 18 blood specimen were drawn from the laboratory confirmed (rK39 diagnostic kit antigen-based dipstick, IT-Leish, DiaMed AG, Switzerland) cases. Enzyme-linked immune-sorbent Assay (ELISA) and PCR testing techniques were used for differential diagnosis to rule out other febrile illness. Of the two tests employed, all (100%) samples were negative for *Yellow Fever*, *Dengue Fever*, *West Nile*, *Chikungunya* and *Rift Valley* Fever viruses by IgM ELISA; *Flavi*, *Alpha*, *Orthobunya*, *Yellow Fever*, *West Nile*, *Dengue*, *Rift Valley Fever* and *Chikungunya* viruses by PCR; *Rickettsia spp*., *Leptospira spp.* and *Salmonella spp*. bacteria and *Plasmodium spp*. parasites by PCR.

### Evaluation of outbreak preparedness and response

A total of seven face to face interviews were conducted among health facility in-charges and Health Management Team (HMT) members. Of the three heath facilities which reported VL cases, one (33%) was a private health care facility, two (66%) had surveillance focal persons, one (33%) had analyzed their data and two (66%) submitted monthly feedback to HMT. All (100%) HMT members and facility in-charges from both private and public facilities were aware of Kala-azar outbreak in Marsabit County. However, VL outbreak and case management sensitization had not been done in all health facilities. Two (66%) health care facilities had been involved in active case finding in the community. Mass screening was conducted on 21st May 2014 at Shurr village where VL confirmed cases were clustered.

Information, Education and Communication (IEC) materials on Kala-azar were not available in all facilities. None of the sampled health facilities had surveillance guidelines for kala-azar, kala-azar standard case definition, emergence and environmental control plan. Only the private health facility (Mountain Clinic) had stocked rK39 diagnosis kit for the last 1 year. None of the health facilities had stocked Kala-azar medication (Sodium stibogluconate (SSG) and paramomycin) in the last 1 year. None of the health care worker in Marsabit County had been trained on Kala-azar case and specimen management in the last 1 year.

## Discussion

One hundred and thirty-six VL cases were confirmed using rK36 rapid diagnostic test (RDT) kit. Blood samples taken for differential diagnosis were negative for several viral, bacterial and parasitic micro-organisms analyzed; *Yellow Fever*, *Dengue Fever*, *West Nile*, *Chikungunya*, *Rift Valley Fever*, *Flavi*, *Alpha*, and *Orthobunya* viruses; *Rickettsia* spp., *Leptospira spp*. and *Salmonella spp.* bacteria and *Plasmodium spp.* parasites.

The first cases (72%) during this outbreak were treated with the Sodium stibogluconate (SSG) monotherapy; the available treatment option at the time of diagnosis. Though efficacious, SSG monotherapy treatment requires a longer hospitalization period (30 days) compared to stibogluconate and paromomycin combined therapy (17 days) [10, 12–15]. However, at the tail end of the outbreak, a combined regimen of sodium stibogluconate and paromomycin was administered. This is the recommended VL first line treatment in Eastern African countries [7, 10].

Males were more affected than females. Although all age categories were affected, majority of the cases were older male between 15 and 44 years. This is inconsistent with a study conducted among the Ugandan and Kenyan Pokot community where the most affected age group were male between 5 and 14 years [8]. This can be attributed to culturally defined duties in these nomadic communities where men of different age groups are involved in cattle herding while women are left at home to conduct household chores. The age group shift from 5 to 14 years to 15–44 years can be attributed to the introduction of free primary education in Kenya. The primary school eligible age group seems to have abandoned herding to schooling. Majority (30%) of the cases were 15–29 years old which is similar to cases reported at Metema Hospital, Ethiopia from 2008 to 2012 [17].

The clinical picture of VL patient in Marsabit differed from those presented among the Pokot communities despite the fact that they share similar Manyatta and pastoralist lifestyle. In the current study, more than a third of the cases presented with fever and a third presented with splenomegaly. This is inconsistent with Mueller et al. [8] study among the Pokot community where all the cases presented with fever and more than two third of the cases presented with splenomegaly.

Visceral leishmaniasis patients’ response to treatment can be clinically evaluated by monitoring reduction of spleen/liver size and examining the normalization of blood cell counts through Full Haemogram test which serves as an indicator of bone marrow recovery [5]. Assessment of the Kidney function (Creatinine levels) is used to monitor toxicity associated with sodium stibogluconate-SSG treatment (ZijlstraEE and el-HassanAM, [22]). In this study no spleen/liver size reduction assessment was conducted while liver function and blood cell count were inconsistently monitored. This is suggestive of poor VL case management.

## Conclusions

The study documents new VL endemic Foci in Marsabit County, where the disease is a big burden to the currently devolved health care department. Cases were inadequately managed. The County outbreak preparedness was inadequate.

## Recommendations

The County health department should initiate one health VL surveillance in both human and animal (cattle, sheep, camels, goats, donkey, dogs and other VL incriminated wild animals) population and carry out periodic VL case management training to assure optimal case management. Public private partnerships should be strengthened in kala-azar diagnosis, surveillance, prevention, management and control.

## Supplementary information

**Additional file 1.** Kala-azar case investigation form.

**Additional file 2.** Kala-azar outbreak preparedness form.

## Data Availability

The de-identified datasets and all supplementary files used and/or analysed during the current study are available from the corresponding author on reasonable request. The de-identified data and all supplementary files will also be deposited in publicly available repositories after manuscript publication.

## Abbreviations

AFENET: Africa Field Epidemiology Network
BSc: Bachelors of Science
DSRU: Disease Surveillance and Response Unit (DSRU)
ELISA: Enzyme-Linked Immunosorbent Assay
FELTP: Field Epidemiology and Laboratory Training Program
FHG: Full haemogram
HCWs: Health care workers
HMT: Health Management Team
IEC: Information, Education and Communication
IgM: Immuno-globulin M
LFTs: Liver Function Tests
MSc: Masters of Science
NPHLs: National Public Health Laboratory services
PCR: Polymerase Chain Reaction
Spp: Species
SSG: Sodium stibogluconate
VL: Visceral leishmaniasis
