# Supplementary material for: Characterization of visceral leishmaniasis outbreak, Marsabit County, Kenya, 2014
Source: BMC Public Health. 2020 Apr 5;20:446. doi: 10.1186/s12889-020-08532-9 (PMC7132962; doi:10.1186/s12889-020-08532-9)
Supplement: Supplementary file 1 — Additional file 1. Kala-azar case investigation form. [file 12889_2020_8532_MOESM1_ESM.doc]

# **KALA-AZAR CASE INVESTIGATION FORM**

*Fill this form for each person who meets the surveillance case definition of suspected_ kala-azar.*

| Serial number: ______________ Reporting facility: ________________  Public Private Other  Name of interviewer________________________  Date__________/_____/___________(DD/MM/YYYYY) | | | | | | | | | | | |
| --- | --- | --- | --- | --- | --- | --- | --- | --- | --- | --- | --- |
| 1. **Patient Information** | | | | | | | | | | | |
| Patient name: | | | | | | | | | | | Sex: {M/F} |
| Patient ID: | | | | | | | | | | | |
| If child, name of the caregiver/parent/guardian: | | | | | | | | | | | |
| Age: ______Years If child less than 1 year old: _______Months | | | | | | | | | | | |
| Occupation: ________________________________________________________________________ | | | | | | | | | | | |
| Residence (7days before illness): | | | | | | | | | | | |
| Village:__________________ | | | | | Sub-Location:________________ | | | | | Location:________________ | |
| Division:__________________ | | | | | Sub-County:_________________ | | | | | County:_________________ | |
| Level of education: None Primary Secondary Tertiary (check highest attained) | | | | | | | | | | | |
| Marital status | | | | Single | | | | |  | | |
|  |  |  |  | Married | | | | | Monogamy polygamy | | |
| **2. Clinical Information** | | | | | | | | | | | |
| *Mark clinical signs and symptoms present* {*Y= Yes N= No*} | | | | | | | | | | | |
| When did you first became ill? :_____/_____/2014 (DD/MM/YYYY)  **Signs and Symptoms** | | | | | | | | | | | |
| Recurrent Fever | | | | | | Y | N |  | | | |
| Chills | | | | | | Y | N |  | | | |
| severe headache | | | | | | Y | N |  | | | |
| Aching muscles | | | | | | Y | N |  | | | |
| Prostration | | | | | | Y | N |  | | | |
| Anorexia | | | | | | Y | N |  | | | |
| Weight loss | | | | | | Y | N |  | | | |
| Splenomegaly | | | | | | Y | N | Physical examination | | | |
| Hepatomegaly | | | | | | Y | N | Physical examination | | | |
| Lymphadenopathy | | | | | | Y | N | Physical examination | | | |
| Yellowing of eyes (jaundice) | | | | | | Y | N |  | | | |
| Anemia | | | | | | Y | N |  | | | |
| hemorrhage | | | | | | Y | N |  | | | |
| respiratory distress syndrome | | | | | | Y | N |  | | | |
| Malnutrition signs | | | | | | Y | N |  | | | |
| Rashes | | | | | | Y | N | If yes Where? Chest Back Arms | | | |
| Dizziness resulting in fainting | | | | | | Y | N |  | | | |
| Have you had similar illness before | | | | | | Y | N | If yes, when (which month and year) _____________ | | | |
| **Laboratory Tests**  Malaria Blood Smear (Positive/Negative): _________________________  Full blood count:  WBC count-_____________________  RBC count-______________________  Platelet count-____________________  HB: _________________________  ESR ___________________________  Renal function test __________________________________________________________________________________________________________________________________________________________________________  Kalaazar Tests:  Rk39 (Positive/Negative) _______________________________________ if positive/negative, draw blood for PCR confirmation...............................................................  Other( specify):______________________ | | | | | | | | | | | |
| Was patient admitted | Y | N | **Outcome : Alive Dead** | | | | | | | | |
| **Treatment history** | | | | | | | | | | | |
| Are you taking any medication? (Oral or injectable). Yes/No: _________________________  If yes, confirm the treatment regimen from record. _________________________________for how many days: ________Days | | | | | | | | | | | |

| **3. Exposure information *(****Probe for exposure to etiologic agent)* | | | | | |
| --- | --- | --- | --- | --- | --- |
| Have you been in contact with anyone with similar illness to yours two weeks prior? | | Y | | N | |
| In recent past have you noticed any increase in mosquitoes near your residence? | | Y | | N | |
| Have you been bitten by mosquitoes in the past 4 months? | | Y | | N | |
| Type of housing: Permanent Stone house Semi-permanent Mud/Grass | | | | | |
| Where do you usually sleep during the day? In the house Outside, Others specify:__________ | | | | | |
| Do you always use a net while sleeping? | Y | | | N | |
| If yes is it a treated net? | Y | | | N | |
| Do you rear livestock? Yes/No ______________ if yes, list them___________________________ | | | | | |
| Do you sleep in the same house/share sleeping space with the livestock? Yes/No _____________________ | | | | | |
| Source of water. (indicate) ______________________________________________________________ | | | | | |
| How do you store your water? Underground water tanks Jerricans Over head tanks | | | | | |
| Did you visit forested areas in the last 2 weeks? (Yes/No) ____________  if yes where?______________________________________ | | | | | |
| Have you notice increased garbage around your residence? | | | | | |
| How do you dispose your solid waste? Dug pits Bins Heaped garbage Burning Haphazard | | | | | |
| Are there bushes around the house? | | | Y | | N |
| Travel history: Have you travelled in the last 6 month | | | Y | | N |
| *If yes in above, Where? Specify___________________ When was this travel? Specify________________* | | | | | |
| **If a school going child then which school do the child attend (specify)____________________________** | | | | | |
