# Supplementary material for: Characterization of visceral leishmaniasis outbreak, Marsabit County, Kenya, 2014
Source: BMC Public Health. 2020 Apr 5;20:446. doi: 10.1186/s12889-020-08532-9 (PMC7132962; doi:10.1186/s12889-020-08532-9)
Supplement: Supplementary file 2 — Additional file 2. Kala-azar outbreak preparedness form. [file 12889_2020_8532_MOESM2_ESM.doc]

# KALA-AZAR OUTBREAK RESPONSE EVALUATION QUESTIONNARE, MARSABIT COUNTY, MAY 2014

**Questionnaire No ________________ Name of interviewer_______________________**

**Name of interviewee ____________________Designation: ___________________________**

1. Name of the Facility____________ Sub-County_______________ Division____________
2. levels of facility (check where appropriate)
   - Level i
   - Level ii
   - Level iii
   - Level vi
   - Level v

3. Type of facility

- public
- private

**ASSESSMENT OF THE OUTBREAK**

1. How did you get to know of the occurrence of the Kala-azar outbreak?

Media reports Community Others Specify------------------------

1. How were the first cases notified to health authorities

- through surveillance officer
- through DMOH
- Other? Specify _______________________________________________

3. On what basis was it decided that this was an outbreak? (check where appropriate)

- A single case?
  - A cluster of cases?
  - Case incidence greater than expected (compared with the same period of time in previous years)?

6. How long did the information take to reach Health Management Team (HMT) from the area when the outbreak occurred? _____________ (Days)

7. What were the first actions the HMT took on getting report of a possible Kala-azar outbreak?

- Telephone call to verify the information
- Constituted a rapid response team
- Dispatch of a rapid-response team
- Alert the surveillance officer
- Other specify______________________________________

**Verification and Response.**

1. How was the diagnosis of kala-azar confirmed?
   - Clinical case definition
   - Laboratory confirmation
   - Epidemiological link with a confirmed case
2. Where was the laboratory diagnosis done________________?
3. How many laboratory officers serve in the laboratory department? _________________
4. How many laboratory staffs are trained in Kala-azar diagnosis in the last one year? ____
5. How long did the laboratory take to provide confirmation?
   - 1 hour
   - 3 hours
   - One day
   - Two days
   - More than week
   - Others specify______________
6. Are you able to correctly interpret the results of kala-azar you get from the Lab?

Yes No

1. Is kala-azar case definition available to HCWs?

Yes No

1. Did you have readily available kits for diagnosis in your facilities? Yes/No __________
2. Did you experience shortage of Kits in the last 3 months?

Yes No

If yes specify____________________________________________________________

1. Have you experienced stock outs of specimen collection containers in the last 3 months? Yes No
2. Did you have challenges/difficulties storing the specimens?

Yes No

If yes specify ____________________________________________________

1. Was transportation of specimen to the referral Lab a challenge?

Yes No

If yes specify ____________________________________________________________

1. Are there enough cool boxes for specimen transportation?

Yes No

**ASSESSMENT OF THE OUTBREAK**

1. Is there a disease outbreak Response committee (Rapid response team)?

Yes No

List members’ designation________________________________________

- - Frequency of meetings _____________
  - Minutes available? Yes No

1. What measures have been taken to control the outbreak

- Insecticide residual spray.
- Clinicians sensitization
- Community sensitization
- Encourage use of mosquito nets
- Draining away stagnant water.
- Others_____________________________________________________________________________________________________________________________

1. How do you monitor the response?
   - Follow-up of the outbreak by epidemiological reports?
   - Impact of control activities on epidemiological trends?
   - Continued line listing of new cases
   - Periodic laboratory testing for kala-azar for suspected cases
   - Others (specify)_____________________________
2. Do you have Kala-azar emergency action plan available?

Yes No

- 1. If yes specify (tick where applicable)
- logistics plan -what is available, what is needed);
- Plan staff responsibilities and assignments
- availability of financial support
- Implementation plan of the control measures
- Others specify……

**Management of Information**

1. Do you have an updated line list of the suspected cases?

Yes No

1. Is the data generated analyzed? Yes/No ________________
2. Is the data analyzed used in making decisions? (verify by examples)

Yes No

1. Is feedback given to:
   1. Community members Yes No
   2. Health care workers? Yes/No __________________
   3. Others stake holders, e.g. private Facilities

Yes No

1. What communicate mobilization strategy did you use?

Chief’s Barraza’s religious centers media others______

**Kala-azar Case management**

1. How many patients have been diagnosed with kala-azar so far? ___________________
2. Any admissions? Yes No

If yes, how many _______________

1. Did the patients receive treatment? Yes No
2. How many clinicians serve this health facility?

A) Doctors _________

B) C.O __________

C) Nursing officers__________

1. How many clinicians have been trained in Kala-azar case management in the last one year? _______________________________________________________________________
2. What treatment were given to the patients?

- Pentavalent antimonials sodium stibogluconate and meglumine antimoniate
- amphotericin B
- Paromomycin
- Sitamaquine
- sodium stibogluconate and paromomycin
- liposomal
- amphotericin B and miltefosine
- Others___________

1. How many pharmacists serve this health facility? _______________________
2. How many pharmacists have been trained in Kala-azar case management in the last one year? _______________________________________________________________________

1. Which VL medications are available in your facility

 Pentavalent antimonials sodium stibogluconate and meglumine antimoniate

 amphotericin B

 Paromomycin

 Sitamaquine

 sodium stibogluconate and paromomycin

 liposomal

 amphotericin B and miltefosine

 Others___________

1. Has there been stock out of Kala-azar medications in the last 3 months? Yes/No _________________________________________________________________________
2. Any health education sessions to patients and family members in the health care facility?

Yes No

1. Are charts illustrating proper management of kala-azar cases prepared and available to health care workers? Yes No

**Environmental control of Kala-azar and community intervention strategy**

1. Do you have an environmental control plan for **Kala-azar? Yes** No

If yes, list control activities conducted

- - 1. Fogging
    2. Distribution of insecticide treated nets
    3. House to house insecticide spraying
    4. Others (specify)

_________________________________________________________________________________________________________________________________________________________

1. Has mapping been done showing sandflies breeding sides in your area? Yes No

If yes do you have a map showing hot spots (verify)

_____________________

1. Are mosquito nets available for distribution in your facility? Yes No
2. Was there active case-finding of **Kala-azar** done in the community

Yes No
